# Supplementary material for: Progressive alterations in mineral contents in citrus genotypes toward Alternaria citri causing brown spot of citrus
Source: PLoS One. 2024 Jul 3;19(7):e0306031. doi: 10.1371/journal.pone.0306031 (PMC11221669; doi:10.1371/journal.pone.0306031)
Supplement: Attachment — Submitted filename: Comments.docx [file pone.0306031.s002.docx]

Commets

Include page numbers and line numbers in the manuscript file. Use continuous line numbers **Abstract**

Line 2: replace the word « crop » with « crops »

Line 2: why do you choose the word « alteration » why not reduction or change of in

mineral content in citrus

line 3: change the word « contents » to content

line 3: replace « after the attack of brown spot disease » with « after brown spot disease attack »

line 3: replace « The leaf sample of susceptible citrus » with « The mineral contents of leaf samples from of susceptible citrus »

line 5: replace « …. were analyzed and significant variations » with « …. were analyzed. significant variation »

line 7: replace «Resistant and Susceptible » with « resistant and susceptible »

Line 7 and 8: replace « The Nested Structured Analysis of variance indicated significant alterations in mineral status (N, P, K, Ca, Mg, and Zn, Na, Fe and Cu) of citrus leaves. » by « The analysis of variance showed significant changes in mineral levels of citrus leaves, including nitrogen (N), phosphorus (P), potassium (K), calcium (Ca), magnesium (Mg), zinc (Zn), sodium (Na), iron (Fe), and copper (Cu). »

Line 9: replace « Resistant type plants showed 6.63% and1.44% while susceptible type indicated 6.07% and 1.19% difference in concentration of N and P respectively. »  with « The results indicate that the concentration of N and P differed by 6.63% and 1.44%, respectively, in resistant plants, while susceptible plants showed a difference of 6.07% and 1.19%. »

Line11: replace «  with « resistant plants showed a higher concentrations of K, Ca, Mg, Zn, Na, Fe, and Cu at 8.40, 2.1, 1.83, 2.21, 1.58, 2.89, and 0.36 ppm respectively, compared to susceptible plants which showed concentrations of 5.99, 1.93, 1.47, 1.09, 1.24, 1.81, and 0.31 ppm respectively. »

Added key words for abstract

**MATERIALS AND METHODES**

**Line 1: replace «** One-year old » with « One-year-old »

Line 2: mention the type of varieties (resistance and susceptible)

Line 5: mention the greenhouse conditions (RH % and Temperature ….)

Line 7: mention the dose of fertilizers  and the amount and frequency of irrigation?

Line 7: replace « no. of irrigations » with « the number of irrigations »

Line 8: plants in a healthy condition. (add « a »)

Line 8: Diseased samples (add « s » to sample)

Line 8: replace « characteristics symptoms » with « characteristic symptoms »

Line 8: « Diseased sample showing characteristics symptoms of brown spot of citrus were collected in brown paper bags » mention the number of days after plantation

Line 8: make space between × and 9.5")

Line 11: replace « For isolation of pathogen PDA » with « For the isolation of the pathogen, PDA »

Line 11: is not necessary to mention the PDA composition

Line 12: change « For pathogenicity » to « For the pathogenicity »

Line 13: did you used distilled water or sterile distilled water?

Line 13: surface with blade. did you used sterile blade?

Line 14: muslin cloth: sterile?

Line 14: replace « hemocytometer » with « heamocytometer »

Line 15: replace « For inoculation, fungal suspension was taken in syringe (23Gx 1") and was injected into midrib and veins of lower surface of leaves [18,19] » with « For inoculation, the fungal suspension was taken in a sterile syringe (23Gx 1") and injected into the midrib and veins of lower surface of leaves [18,19] »

Line 16: replace (when max. no. of stomata) with (when maximum number of stomata)

Line 17: Disease incidence was confirmed: mention the period of incubation

Line 18: uninoculated or un-inoculated : homogenized this word in the manuscript

**Sample preparation for the analysis of mineral contents**

Line 1: replace “Plant samples” with “leaves samples”

Line 1: delete (from both inoculated and uninoculated cultivars) is already mentioned

Line 2: “were collected from the greenhouse, Department of Plant Pathology near Centre for Advanced Studies, University of Agriculture Faisalabad. These samples were brought to citrus pathology lab for further process.”

delete “from the greenhouse, Department of Plant Pathology near Centre for Advanced Studies, University of Agriculture Faisalabad” the text will be “were collected and brought to Citrus Pathology Laboratory for further process”

Line 4: replace “sample was subjected to oven (Memmert: UN 30) drying at 65ºC “with “samples were dried in an oven (Memmert: UN 30) at 65 ºC “

Line 4: why 4hrs? Is four hours enough time to dry citrus leaves??

Line 6: change each samples to each sample

Line 7: (RT2-230V).After cooling: add a space

Line 7: replace “After cooling, dilution of suspension was done with distilled

water and later on was examined for the estimation of mineral contents [20] through [21] method and data was analyzed by using Nested structure des” with “After cooling, the suspension was diluted with distilled water and examined for estimation of mineral contents [20, 21]. The data was analyzed using Nested Structure design”

**Estimation of phosphorus (P) from the inoculated and un-inoculated citrus plant leaves**

-This paragraph is not clear. Please make it more comprehensible. Additionally, please include the concentration or molarity of the acids and solutions used.

**Line 1: replace “**Sample solution” with “A sample solution”

Line 3: replace “Then distilled water (reagent black) was used as a substitute of sample solution and absorbance was taken at a wavelength of 720nm” with “Distilled water was used as a substitute for the sample solution to serve as a control. Absorbance was taken at a wavelength of 720nm”

**Estimation of potassium (K) and sodium (Na) from inoculated and uninoculated leaves of citrus plants**

Line 2: replace “following the [23] method” with “following the Official methods of analysis [23]”

**Estimation of magnesium (Mg), calcium (Ca) copper (Cu), zinc (Zn) and iron (Fe) from**

**the inoculated and un-inoculated leaves of citrus**

Line 2: (Atomic Absorption Spectrometer).by using the standards …. Remove the point (.) and add the machine make.

**Estimation of nitrogen/ crude protein from the inoculated and un-inoculated leaves citrus plants**

Line 2: Long necked flask was used to place calculated amount of sample previously oven

dried (D6450, Heraeus): Not clear??

line 4: concentrated H2SO4 were: add the concentration or molarity

line 4: replace “were added and sample was subjected to” with “were added to necked flask. the mixture was subjected to ... »

Line 11: add the reference of Nitrogen (%) and Crude Protein

**Statistical analysis**

Line 4: **“**Leaves sample from inoculated and un-inoculated varsities were collected from the Research Area, Department of Plant Pathology, near CAS (Center of Advanced Studies), University of Agriculture Faisalabad (UAF).” Remove this text

Line 8: PROC MIXED: add more details (Software, version, ANOVA, level of significance 5%, 1% or 0.01% …….

**Results**

This section requires clarification to make it easy to understand.

The tables are too cluttered. It is better to simplify and verify them.

Line 6 and line 27: “percent” Use % (check all the manuscript)

Line 35: “. Un-inoculated (2.30ppm) and inoculated ….” Remove the point

Line 50: replace “  P<0.05 as shown by Table 3 and Table 4.” With “P<0.05 as shown in Table 3 and Table 4.”

For table: For the table, please provide the meanings of any abbreviations used. For example, SOV and DF

It is better to make the value of each parameter separately. For instance, show the nitrogen value for each variety of citrus leaves, both inoculated and non-inoculated, to make a comparison (table 2).

Table 2: Verify the value for “Un-Inoculated ” is 8.56 or 8.72?

**Discussion**

“Such plants are more susceptible to attack of pathogens as indicated by [29].” Cite the author’s name

“which is also witnessed by [34, 35]. who described that N contents are reduced after attack of fungi?” Cite the author’s name

“inoculated leaves of citrus plants which is also witnessed by [34, 35]”. Cite the author’s name. check all references
