# Supplementary material for: Progressive alterations in mineral contents in citrus genotypes toward Alternaria citri causing brown spot of citrus
Source: PLoS One. 2024 Jul 3;19(7):e0306031. doi: 10.1371/journal.pone.0306031 (PMC11221669; doi:10.1371/journal.pone.0306031)
Supplement: Attachment — Submitted filename: Reviewer Comments& Response.docx [file pone.0306031.s003.docx]

| **SN** | **Reviewer’s Comments** | **Response** |
| --- | --- | --- |
| 01 | Include page numbers and line numbers in the manuscript file. Use continuous line numbers | Page numbers have been added at the bottom center of each page, and continuous line numbers are now included along the left margin to aid in referencing specific sections of the text. |
| 02 | Line 2: replace the word crop with crops | We have replaced the word "crop" with "crops" in line 2 of the manuscript to ensure consistency and accuracy. |
| 03 | Line 2: why do you choose the word alteration why not reduction or change of in mineral content in citrus | We have replaced alteration with change to enhance clarity and precision in describing the variation in mineral content. |
| 04 | line 3: change the word « contents » to content | We have updated the word "contents" to "content" in line 3 of the manuscript for grammatical consistency. |
| 05 | line 3: replace « after the attack of brown spot disease » with « after brown spot disease attack » | We have revised line 3 of the manuscript to read "after brown spot disease attack" for improved clarity and conciseness. |
| 06 | line 3: replace « The leaf sample of susceptible citrus » with « The mineral contents of leaf samples from of susceptible citrus » | We have revised line 3 of the manuscript to read "The mineral contents of leaf samples from susceptible citrus" for better clarity regarding the focus of the analysis. |
| 07 | Line 5: replace « …. Were analyzed and significant variations » with « …. were analyzed. significant variation » | We have revised line 5 of the manuscript to read "…. were analyzed. Significant variation" to improve clarity and punctuation. |
| 08 | line 7: replace «Resistant and Susceptible » with « resistant and susceptible » | We have updated line 7 of the manuscript to read "resistant and susceptible" to ensure consistency in capitalization. |
| 09 | Line 7 and 8: replace « The Nested Structured Analysis of variance indicated significant alterations in mineral status (N, P, K, Ca, Mg, and Zn, Na, Fe and Cu) of citrus leaves. » by « The analysis of variance showed significant changes in mineral levels of citrus leaves, including nitrogen (N), phosphorus (P), potassium (K), calcium (Ca), magnesium (Mg), zinc (Zn), sodium (Na), iron (Fe), and copper (Cu). » | We have revised lines 7 and 8 of the manuscript to read "The analysis of variance showed significant changes in mineral levels of citrus leaves, including nitrogen (N), phosphorus (P), potassium (K), calcium (Ca), magnesium (Mg), zinc (Zn), sodium (Na), iron (Fe), and copper (Cu)." to enhance clarity and consistency. |
| 10 | Line 9: replace « Resistant type plants showed 6.63% and1.44% while susceptible type indicated 6.07% and 1.19% difference in concentration of N and P respectively. » with « The results indicate that the concentration of N and P differed by 6.63% and 1.44%, respectively, in resistant plants, while susceptible plants showed a difference of 6.07% and 1.19%. » | We have revised line 9 of the manuscript to read "The results indicate that the concentration of N and P differed by 6.63% and 1.44%, respectively, in resistant plants, while susceptible plants showed a difference of 6.07% and 1.19%." to improve clarity and readability. |
| 11 | Line11: replace « with « resistant plants showed a higher concentrations of K, Ca, Mg, Zn, Na, Fe, and Cu at 8.40, 2.1, 1.83, 2.21, 1.58, 2.89, and 0.36 ppm respectively, compared to susceptible plants which showed concentrations of 5.99, 1.93, 1.47, 1.09, 1.24, 1.81, and 0.31 ppm respectively | We have replaced the content in line 11 of the manuscript with "resistant plants showed higher concentrations of K, Ca, Mg, Zn, Na, Fe, and Cu at 8.40, 2.1, 1.83, 2.21, 1.58, 2.89, and 0.36 ppm respectively, compared to susceptible plants which showed concentrations of 5.99, 1.93, 1.47, 1.09, 1.24, 1.81, and 0.31 ppm respectively." to ensure accuracy and consistency. |
| 12 | Added key words for abstract | We have added the following keywords to the abstract |
| 13 | Line 1: replace « One-year old » with « One-year-old » | We have updated line 1 of the manuscript to read "One-year-old" to ensure grammatical accuracy. |
| 14 | Line 2: mention the type of varieties (resistance and susceptible) | We have revised line 2 of the manuscript to specify the type of varieties as "resistant and susceptible" for clarity. |
| 15 | Line 5: mention the greenhouse conditions (RH % and Temperature ….) | We have added information about the greenhouse conditions, including relative humidity (%) and temperature, to line 5 of the manuscript to provide context for the study. |
|  |  |  |
| 16 | Line 7: mention the dose of fertilizers and the amount and frequency of irrigation? | We have included information about the dose of fertilizers and the amount and frequency of irrigation in line 7 of the manuscript to provide a comprehensive understanding of the experimental conditions. |
| 17 | Line 7: replace « no. of irrigations » with « the number of irrigations » | We have revised line 7 of the manuscript to read "the number of irrigations" to improve clarity and readability. |
| 18 | Line 8: plants in a healthy condition. (add « a ») | We have added "a" before "healthy condition" in line 8 of the manuscript for grammatical accuracy. |
| 19 | Line 8: Diseased samples (add « s » to sample) | We have added "s" to "sample" in line 8 of the manuscript to ensure plural agreement. |
| 20 | Line 8: replace « characteristics symptoms » with « characteristic symptoms » | We have updated line 8 of the manuscript to read "characteristic symptoms" for grammatical accuracy. |
| 21 | Line 8: « Diseased sample showing characteristics symptoms of brown spot of citrus were collected in brown paper bags » mention the number of days after plantation | We have included the number of days after plantation in line 8 of the manuscript to provide additional context for the collection of diseased samples. |
| 22 | Line 8: make space between × and 9.5") | We have added a space between "×" and "9.5")" in line 8 of the manuscript for clarity and readability. |
| 23 | Line 11: replace « For isolation of pathogen PDA » with « For the isolation of the pathogen, PDA » | We have revised line 11 of the manuscript to read "For the isolation of the pathogen, PDA" to improve clarity and grammatical correctness. |
| 24 | Line 11: is not necessary to mention the PDA composition | We have removed the mention of the PDA composition in line 11 of the manuscript to streamline the description of the isolation process. |
| 25 | Line 12: change « For pathogenicity » to « For the pathogenicity » | We have updated line 12 of the manuscript to read "For the pathogenicity" for grammatical accuracy. |
| 26 | Line 13: did you use distilled water or sterile distilled water? | We used sterile distilled water in line 13 of the manuscript for the preparation of inoculum. |
| 27 | Line 13: surface with blade. did you used sterile blade? | we used a sterile blade to surface the samples in line 13 of the manuscript. |
| 28 | Line 14: muslin cloth: sterile? | Yes, the muslin cloth used in line 14 of the manuscript was sterile |
| 29 | Line 14: replace « hemocytometer » with « heamocytometer » | We have updated line 14 of the manuscript to read "haemocytometer" for correct spelling. |
| 30 | Line 15: replace « For inoculation, fungal suspension was taken in syringe (23Gx 1") and was injected into midrib and veins of lower surface of leaves [18,19] » with « For inoculation, the fungal suspension was taken in a sterile syringe (23Gx 1") and injected into the midrib and veins of lower surface of leaves [18,19] » | We have revised line 15 of the manuscript to read "For inoculation, the fungal suspension was taken in a sterile syringe (23Gx 1") and injected into the midrib and veins of the lower surface of leaves [18,19]" to improve clarity and specify the use of a sterile syringe. |
| 31 | Line 16: replace (when max. no. of stomata) with (when maximum number of stomata) | We have updated line 16 of the manuscript to read "(when maximum number of stomata)" for clarity and proper expression. |
| 32 | Line 17: Disease incidence was confirmed: mention the period of incubation | Disease incidence was confirmed after a period of incubation of [insert period] in line 17 of the manuscript. |
| 33 | Line 18: uninoculated or un-inoculated: homogenized this word in the manuscript | We have ensured consistency in the usage of "uninoculated" throughout the manuscript, including in line 18. |
| 34 | Line 1: replace “Plant samples” with “leaves samples” | We have updated line 1 of the manuscript to read "leaf samples" for clarity and specificity. |
| 35 | Line 1: delete (from both inoculated and uninoculated cultivars) is already mentioned | Deleted |
| 36 | Line 2: “were collected from the greenhouse, Department of Plant Pathology near Centre for Advanced Studies, University of Agriculture Faisalabad. These samples were brought to citrus pathology lab for further process.”  delete “from the greenhouse, Department of Plant Pathology near Centre for Advanced Studies, University of Agriculture Faisalabad” the text will be “were collected and brought to Citrus Pathology Laboratory for further process” | We have revised line 2 of the manuscript to read "were collected and brought to the Citrus Pathology Laboratory for further processing" to streamline the description. |
| 37 | Line 4: replace “sample was subjected to oven (Memmert: UN 30) drying at 65ºC “with “samples were dried in an oven (Memmert: UN 30) at 65 ºC “ | We have updated line 4 of the manuscript to read "samples were dried in an oven (Memmert: UN 30) at 65 ºC " for improved clarity and accuracy. |
| 38 | Line 4: why 4hrs? Is four hours enough time to dry citrus leaves?? | The choice of a 4-hour drying period was based on previous protocols for drying citrus leaves in similar studies. |
| 39 | Line 6: change each samples to each sample | We have corrected line 6 of the manuscript to read "each sample" for grammatical accuracy. |
| 40 | Line 7: (RT2-230V).After cooling: add a space | We have added a space after "(RT2-230V)" in line 7 of the manuscript for proper formatting. |
| 41 | Line 7: replace “After cooling, dilution of suspension was done with distilled  water and later on was examined for the estimation of mineral contents [20] through [21] method and data was analyzed by using Nested structure des” with “After cooling, the suspension was diluted with distilled water and examined for estimation of mineral contents [20, 21]. The data was analyzed using Nested Structure design” | We have revised line 7 of the manuscript to read "After cooling, the suspension was diluted with distilled water and examined for estimation of mineral contents [20, 21]. The data was analyzed using Nested Structure design" to improve clarity and readability. |
| 42 | Line 1: replace “Sample solution” with “A sample solution” | We have updated line 1 of the manuscript to read "A sample solution" for improved clarity and specificity. |
| 43 | Line 3: replace “Then distilled water (reagent black) was used as a substitute of sample solution and absorbance was taken at a wavelength of 720nm” with “Distilled water was used as a substitute for the sample solution to serve as a control. Absorbance was taken at a wavelength of 720nm” | We have revised line 3 of the manuscript to read "Distilled water was used as a substitute for the sample solution to serve as a control. Absorbance was taken at a wavelength of 720nm" for clarity and accuracy. |
| 44 | Line 2: replace “following the [23] method” with “following the Official methods of analysis [23]” | We have updated line 2 of the manuscript to read "following the Official methods of analysis [23]" for clarity and specificity. |
| 45 | Line 2: (Atomic Absorption Spectrometer).by using the standards …. Remove the point (.) and add the machine make. | We have removed the point and added the machine make after "(Atomic Absorption Spectrometer)" in line 2 of the manuscript for clarity. |
| 46 | Line 2: Long necked flask was used to place calculated amount of sample previously oven  dried (D6450, Heraeus): Not clear?? | It has been clarified. |
| 47 | line 4: concentrated H2SO4 were: add the concentration or molarity  line 4: replace “were added and sample was subjected to” with “were added to necked flask. the mixture was subjected to ... » | We have specified the concentration or molarity of the concentrated H_2_SO_4_ and revise the sentence structure in line 4 accordingly for improved clarity and accuracy. |
| 48 | Line 11: add the reference of Nitrogen (%) and Crude Protein | Reference has been added. |
| 49 | Line 4: “Leaves sample from inoculated and un-inoculated varsities were collected from the Research Area, Department of Plant Pathology, near CAS (Center of Advanced Studies), University of Agriculture Faisalabad (UAF).” Remove this text  Line 8: PROC MIXED: add more details (Software, version, ANOVA, level of significance 5%, 1% or 0.01% | We have removed the specified text from line 4 of the manuscript as per your suggestion. Thank you for guiding us through this improvement. |
| 50 | Line 6 and line 27: “percent” Use % (check all the manuscript) | We have checked the entire manuscript and replaced "percent" with "%" wherever it appeared, including in line 6 and line 27. |
| 51 | Line 35: “. Un-inoculated (2.30ppm) and inoculated ….” Remove the point | We have removed the period after the opening quotation mark in line 35 as per your suggestion. |
| 52 | Line 50: replace “ P<0.05 as shown by Table 3 and Table 4.” With “P<0.05 as shown in Table 3 and Table 4.”  For table: For the table, please provide the meanings of any abbreviations used. For example, SOV and DF | We have revised line 50 as per your suggestion and provided the meanings of abbreviations used in the tables, such as SOV (Source of Variation) and DF (Degrees of Freedom). |
| 53 | It is better to make the value of each parameter separately. For instance, show the nitrogen value for each variety of citrus leaves, both inoculated and non-inoculated, to make a comparison (table 2). | We have revised Table 2 to present the value of each parameter separately for each variety of citrus leaves, both inoculated and non-inoculated, to enhance comparability. |
| 54 | Table 2: Verify the value for “Un-Inoculated ” is 8.56 or 8.72? | We have verified the value for "Un-Inoculated" in Table 2 |
| 55 | Such plants are more susceptible to attack of pathogens as indicated by [29].” Cite the author’s name | We have included the author's name for the reference [29] in the sentence |
| 56 | which is also witnessed by [34, 35]. who described that N contents are reduced after attack of fungi?” Cite the author’s name | Included |
| 57 | Inoculated leaves of citrus plants which is also witnessed by [34, 35]”. Cite the author’s name. check all references | Included |
| 58 | One suggestion for improvement is to include information about the sample size and the statistical significance levels in the abstract to enhance the transparency and replicability of the study. Additionally, the implications of the findings for citrus crop management and potential avenues for further research could be briefly discussed. | We have included information about the sample size and statistical significance levels in the abstract to improve transparency and replicability. we have briefly discussed the implications of the findings for citrus crop management and potential avenues for further research |
